# Two Distinct Phonon Wave Effects Control Thermal Transport across the Coherent–Incoherent Regime in Superlattices

**DOI:** 10.1002/advs.202517251

**Published:** 2026-02-04

**Authors:** Jin Yang, Jingyi Zhu, Alan J. H. McGaughey, Wee‐Liat Ong

**Affiliations:** ^1^ ZJUI Institute College of Energy Engineering Zhejiang University, Haining Jiaxing Zhejiang China; ^2^ Department of Mechanical Engineering Carnegie Mellon University Pittsburgh Pennsylvania USA; ^3^ State Key Laboratory of Clean Energy Utilization Zhejiang University Hangzhou Zhejiang China

**Keywords:** coherent phonon, interfaces, multilayers, particle‐wave duality, Wigner transport equation

## Abstract

Superlattices composed of nanometer‐thick constituent layers with smooth interfaces exhibit a minimum in their cross‐plane thermal conductivity as the period thickness is increased, marking a transition from coherent to incoherent phonon transport. Previous attempts to explain this minimum using the phonon Boltzmann transport equation (BTE) required an ad hoc diffuse interface scattering model due to the BTE's inherent particle‐based framework. We apply the phonon Wigner transport equation (WTE) to study superlattices with smooth interfaces, a framework that inherently includes both the particle‐like (i.e., population‐channel) and wave‐like (i.e., coherence‐channel) contributions to thermal conductivity. Our results reveal that the WTE coherence channel is responsible for the thermal conductivity increase in the incoherent regime. The two distinct phonon wave effects in superlattices—the coherent transport induced by wave interference at the interfaces and the WTE coherence‐channel transport enabled by tunneling between phonon modes—are examined in detail, along with their connection to the interfacial vibrational modes.

## Graphical abstract

Thermal transport in smooth‐interface superlattices is studied using the phonon Wigner transport equation, which captures both particle‐like and wave‐like contributions. A minimum in cross‐plane thermal conductivity emerges with increasing period thickness, signaling a crossover from coherent to incoherent phonon transport. The author show that coherent phonons dominate particle‐like transport, while incoherent phonons primarily contribute via the wave‐like channel.

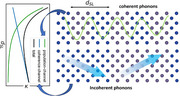

## 1 Introduction

Superlattices have demonstrated significant potential in applications such as solar cells [1], superconductors [2], and microelectronics [3] owing to their peculiar electronic and phonon properties, which are a result of the higher‐order periodicity induced by the layered structure [4, 5]. Controlling thermal transport through nanoscale structural engineering is exceedingly important for the development and improvement of these devices. The hierarchical lattice structure in superlattices offers a controllable approach to modulating phonon transport via the period thickness of the constituents, with marginal impact on their emergent electronic properties [4, 6]. Past experiments on various superlattice systems have revealed a minimum in the cross‐plane thermal conductivity as the period thickness is increased [7, 8, 9, 10, 11, 12]. This minimum thermal conductivity signifies the transition from the coherent regime (corresponding to short period thicknesses) of phonon transport to the incoherent regime (associated with long period thicknesses) [4, 5, 13, 14]. In the coherent regime, wave interference at the interfaces generates coherent phonons that contribute to thermal transport. In the incoherent regime, where the period thickness exceeds the phonon mean free paths (MFPs), however, the intrinsic phonons of the individual layers are mostly relevant [4, 5, 13, 14, 15].

The incoherent regime is typically described using the intrinsic phonons of the individual layers and an empirical model for the interface scattering [16]. On the other hand, the coherent regime in a superlattice with smooth interfaces is commonly modeled by taking one superlattice period as the unit cell [17]. To include these two distinct thermal transport regimes in a single framework, Simkin and Mahan introduced a phonon Boltzmann transport equation (BTE)‐based phenomenological model, where they added an imaginary component to the wave vector to account for the phonon MFP [18]. This model was further refined by Yang and Chen to include the diffuse interface scattering caused by interfacial disorder to better match experimental results [19]. Further developments in computational theory and capabilities have enabled phonon BTE calculations using first‐principles‐based lattice dynamics on superlattices, which incorporate three‐phonon scattering of the coherent phonons. While this approach can capture the thermal conductivity decrease in the coherent regime, it cannot reproduce the thermal conductivity minimum (and thus the thermal conductivity rise in the incoherent regime) unless a diffuse interface scattering model is included [20, 21].

In contrast, molecular dynamics (MD) simulations of thermal transport in superlattices, using non‐equilibrium and Green‐Kubo methods, have predicted the thermal conductivity minima when the interfaces are smooth [22, 23, 24, 25, 26, 27, 28, 29, 30, 31]. Moreover, when interfacial disorder, lattice mismatch, or a random arrangement of atomic layers are included, the coherent phonons are disrupted, which leads to a monotonic increase in thermal conductivity with increasing period thickness [22, 23, 24, 27, 29, 30, 32, 33, 34].

The inconsistency between coherent phonon‐based BTE results and MD simulation results, where no assumptions are made about the nature of the thermal transport in the latter, could arise from the purely particle‐like treatment of phonons in the BTE. In contrast, the recently‐proposed phonon Wigner transport equation (WTE) incorporates the wave‐particle duality of phonons from [35, 36]:

(1) $$\begin{array}{ccc} \;\;\kappa^{\alpha \beta } & = & \frac{1}{V}\;\sum_{q,s,s^{\prime }}\frac{\omega {\left(q\right)}_{s}+\omega {\left(q\right)}_{s^{\prime }}}{4}\left[\frac{C{\left(q\right)}_{s}}{\omega {\left(q\right)}_{s}}+\frac{C{\left(q\right)}_{s^{\prime }}}{\omega {\left(q\right)}_{s^{\prime }}}\right]\\ & & \times v^{\alpha }{\left(q\right)}_{s,s^{\prime }}v^{\beta }{\left(q\right)}_{s^{\prime },s}\frac{\left[\Gamma {\left(q\right)}_{s}+\Gamma {\left(q\right)}_{s^{\prime }}\right]/2}{{\left[\omega {\left(q\right)}_{s^{\prime }}-\omega {\left(q\right)}_{s}\right]}^{2}+{\left[\Gamma {\left(q\right)}_{s}+\Gamma {\left(q\right)}_{s^{\prime }}\right]}^{2}/4} \end{array}$$

where κ and *V* are the thermal conductivity and system volume, ***q*** indexes the wave vector, *s* and *s*′ label phonon modes, *ω*(***q***)_*s*_, *C*(***q***)_*s*_ and Γ(***q***)_*s*_ denote the mode frequency, specific heat, and linewidth, respectively, and *v* ^α^(***q***)_*s*,*s*′_ represents the smooth‐phase group velocity matrix of a mode pair along the Cartesian α‐direction. The population‐channel (κ_*p*_) corresponds to the diagonal terms (*s*  = *s*′), capturing the particle‐like propagation of single phonon modes, while the coherence‐channel (κ_*c*_) arises from the off‐diagonal terms ($\left(s\neq s^{\prime }\right)$), reflecting the wave‐like tunneling between two phonon modes [35, 36]. The WTE offers a promising framework to investigate phonon transport mechanisms in materials with strong anharmonicity, such as perovskites [37, 38] and Zintl compounds [39], as well as in systems with large unit cells, including amorphous silicon [40] and LaPO_4_‐based alloys [41]. It thus opens new avenues for elevating our understanding of superlattices. We emphasize that the terminology “coherence‐channel” in the WTE should not be confused with the “coherent” phonons in a superlattice. The former refers to the wave‐like tunneling transport of phonons, while the latter indicates the wave interference at interfaces between the intrinsic phonons of the individual layers in a superlattice. To avoid ambiguity, the term “wave‐channel” is used herein instead of “coherence‐channel.”

In this study, two superlattice systems with smooth interfaces are studied: (i) Lennard‐Jones (LJ) potential‐based Ar and heavy Ar (hAr) superlattices with a mass mismatch of *m*_hAr_ = 2*m*_Ar_ (i.e., Ar[*N*]hAr[*N*]), and (ii) first‐principles‐based Si[*N*]Ge[*N*] superlattices, where *N* indicates the period thickness in unit cells. We first demonstrate that the phonon WTE results for these superlattice systems predict the minimum thermal conductivity. The thermal conductivity rise in the incoherent regime is revealed to stem entirely from the wave‐channel contributions. Next, we explore the relationship between the population‐/wave‐channel transport and coherent transport by analyzing the phonon modal characteristics, discovering that population‐channel contributions are primarily associated with coherent phonons. Finally, we investigate the vibrational properties near the interfaces and their connection to phonon transport in the superlattice. Our results highlight that coherent phonons are facilitated by interface‐localized atomic vibrations and possess high group velocities, explaining why these phonons predominantly contribute to population‐channel thermal transport.

## 2 Results and Discussion

### 2.1 Reproduction of Minimum Thermal Conductivity Using the Phonon Wigner Transport Equation

**FIGURE 1:**
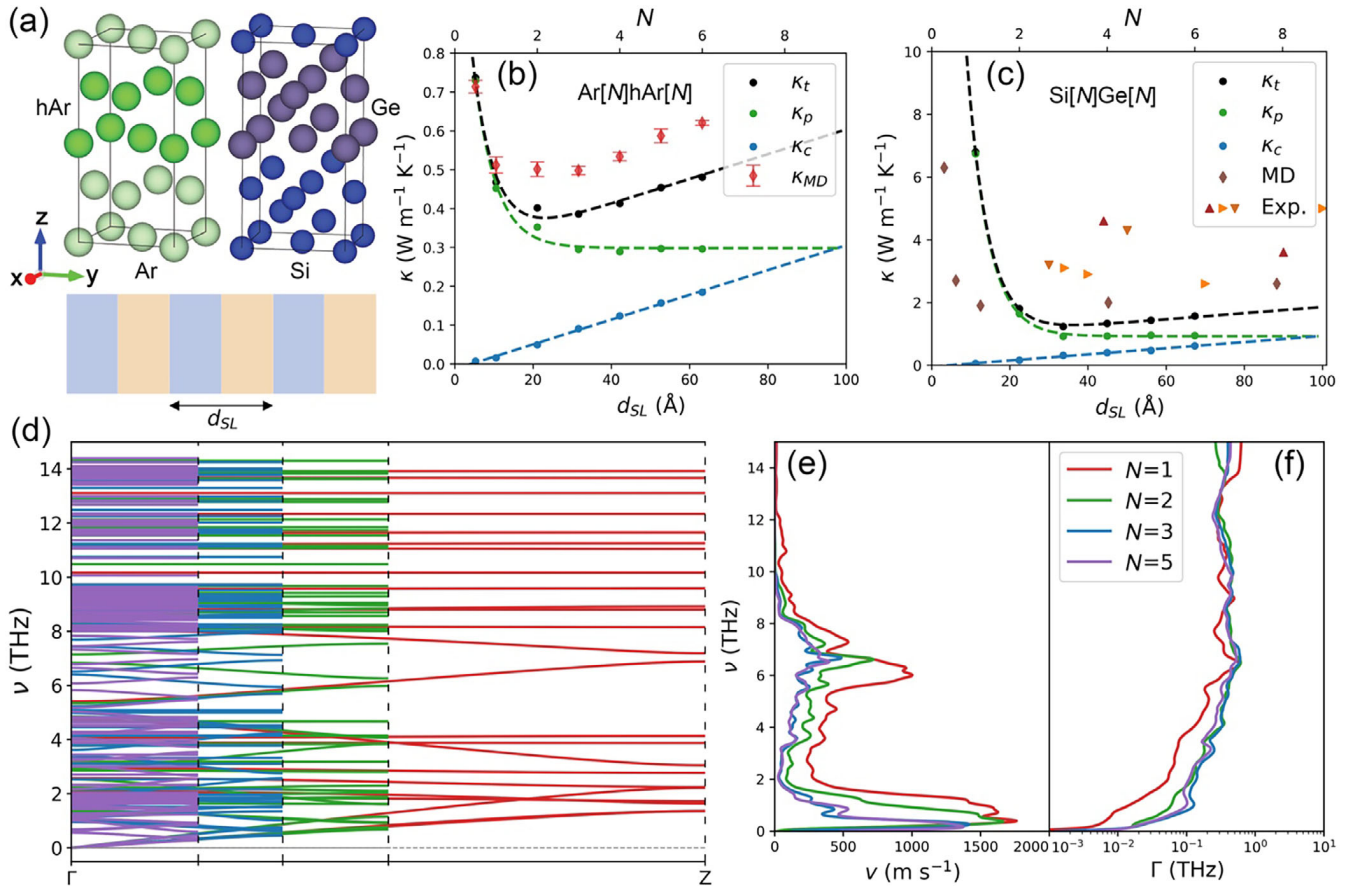
Structures and thermal transport properties of two superlattice systems. (a) The top two pictures show the unit cells of the Ar[1]hAr[1] and Si[1]Ge[1] superlattices. Each period contains two unit cells, one from each constituent. The bottom picture illustrates the superlattice structure, with *d_SL_* denoting the period thickness. Cross‐plane (i.e., along the Cartesian *z*‐direction) thermal conductivity of (b) Ar[*N*]hAr[*N*] superlattices and (c) Si[*N*]Ge[*N*] superlattices vs. *d_SL_* (bottom axis) and *N* (top axis). κ_*t*_, κ_*p*_, and κ_*c*_ represent the total WTE thermal conductivity and its decomposition into the population‐channel and wave‐channel contributions. The dashed lines are an exponential decay fit to κ_*p*_, a linear fit to κ_*c*_, and their summation. κ_*MD*_ in (b) denotes the non‐equilibrium MD thermal conductivity with error bars derived from the standard deviation of three independent runs. The MD [42] and Exp [43, 44, 45]. in (c) correspond to data extracted from the literature. For the Si[*N*]Ge[*N*] superlattices with *N* = 1, 2, 3, and 5: (d) Phonon dispersions where Γ and Z represent the (0 0 0) and (0 0 0.5) points in the Brillouin zone, (e) frequency‐dependent average group velocity along the cross‐plane direction, and (f) frequency‐dependent average scattering rates.

The structures and optimized unit cells of the Ar[1]hAr[1] and Si[1]Ge[1] superlattices with smooth interfaces are shown in Figure 1a. The lattice constants are *a* = *b* = 5.27 Å and *c* = 10.54 Å for Ar[1]hAr[1], and *a* = *b* = 5.61 Å and *c* = 11.22 Å for Si[1]Ge[1]. The total cross‐plane thermal conductivities from the WTE, along with its population‐channel and wave‐channel contributions, are plotted in Figure 1b as a function of the period thickness (*d_SL_*) for the Ar[*N*]hAr[*N*] superlattices at a temperature of 20 K. The WTE predicts a minimum in thermal conductivity, a finding that is consistent with our nonequilibrium MD results, whereas the difference in magnitude likely arises from the use of classical statistics in the MD simulations. The population‐channel thermal conductivity drops exponentially in the coherent regime and then plateaus, consistent with prior BTE predictions [21]. Interestingly, the increase in thermal conductivity in the incoherent regime is entirely driven by the linear growth in the wave‐channel thermal conductivity. A similar trend is observed in the Si[*N*]Ge[*N*] superlattices at a temperature of 300 K (Figure 1c), indicating that the Ar[*N*]hAr[*N*] results are not specific to a particular system. Our computed thermal conductivities for Si[*N*]Ge[*N*] superlattices generally align well with previous MD simulations [42] and experimental measurements [43, 44, 45]. Any discrepancies are likely due to the variations in the employed potentials and structures.

To gain further insights, the phonon properties of the Si[*N*]Ge[*N*] superlattices are examined for the rest of the paper. As the period thickness increases, the breakdown of the initial translational symmetry induces band folding in the phonon dispersions (Figure 1d) [46]. This folding produces minibands with gaps at the newly‐formed Brillouin zone edges, which reduce the group velocities. The reduction is largest near these edges. After averaging over the wave vector at each frequency (ν), the group velocities converge to an *N*‐independent value after *N* = 3, which coincides with the location of the thermal conductivity minimum (Figure 1c,e). As shown in Figure 1f, the wave‐vector averaged frequency‐dependent scattering rates, Γ, initially increase with increasing *N* before plateauing, a trend consistent with that observed in AlN/GaN superlattices [47]. On one hand, the variations in the group velocities and scattering rates explain the decrease in κ_*p*_ at short period thickness, and their convergence accounts for the constant κ_*p*_ observed at larger period thicknesses. On the other hand, the reduced inter‐band spacing and higher converged scattering rate enhance the overlap of linewidths across different bands, thereby driving the rise in κ_*c*_  [37].

### 2.2 Connection Between Phonon Coherent Transport and Population‐/Wave‐Channel Transport

**FIGURE 2:**
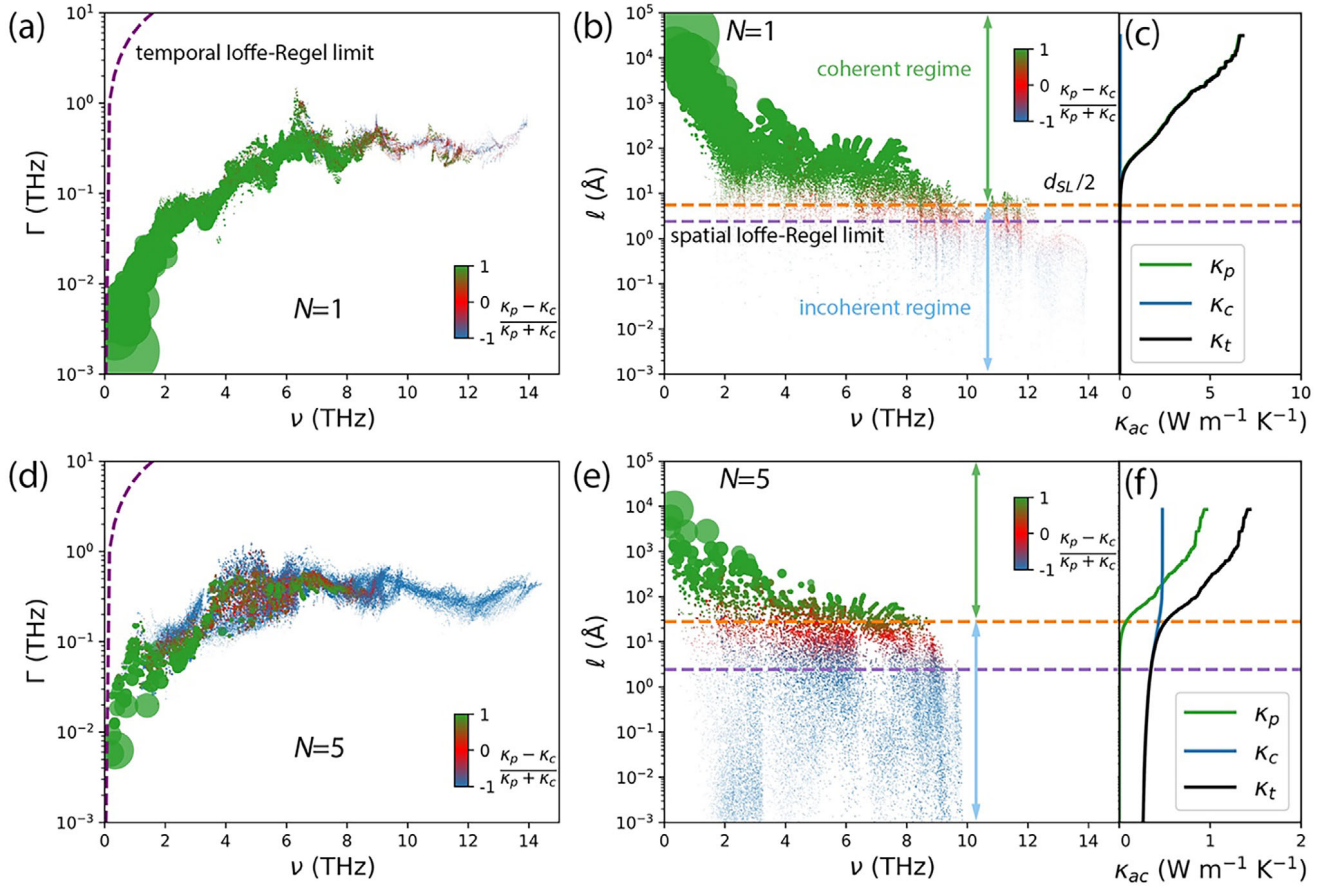
(a) Phonon scattering rate and (b) MFP as a function of phonon frequency, with (c) MFP‐dependent accumulative thermal conductivity for the Si[*N*]Ge[*N*] superlattice with *N* = 1. (d–f) are the same as (a–c) but for *N* = 5. The area of each circle is proportional to the contribution of that mode to the total thermal conductivity. The circle's color indicates the origin of its thermal conductivity: green for 100% from population‐channel; blue for 100% from wave‐channel; and red for equal contributions from both channels. The graded colors represent a varying percentage from each channel. The purple dashed lines in (a, d) correspond to the temporal Ioffe‐Regel limit, defined as Γ_*IR*_ (ν) =  2πν, while those in (b, c, e, f) indicate the spatial Ioffe‐Regel limit, defined as the average atomic distance. The orange dashed lines in (b, c, e, f) mark the half‐period thickness, which approximates the spacing between neighboring interfaces.

We now further elucidate the mode‐level details for κ_*p*_ and κ_*c*_, with the results shown in Figure 2. The phonon scattering rates are plotted vs. frequency in Figure 2a (*N* = 1) and 2d (*N* = 5). All phonon modes are below the Ioffe‐Regel temporal limit [defined as Γ_*IR*_(ν) = 2πν], validating their quasi‐particle treatment [35, 48]. The marker color indicates how the population channel (green) and wave channel (blue) contribute to that mode's thermal conductivity, with red indicating an equal contribution from each channel. A higher proportion of modes contributes primarily through the population‐channel for *N* = 1 than for *N* = 5. For *N* = 1, a negligible portion of high‐frequency modes (>8 THz) contribute primarily through the wave channel (see the frequency‐dependent accumulative thermal conductivity plotted in Figure S2a), whereas for *N* = 5, modes above 2 THz start to contribute to the wave channel (Figure S2b).

Different methods have been proposed to distinguish the contributions of coherent phonons and incoherent phonons to the thermal conductivity of a superlattice [24, 26, 32, 49, 50]. We select the interface spacing (i.e., half of the period thickness, *d_SL_*/2), which is a commonly used feature in previous studies [18, 26, 50], as the length scale for comparing to the MFP to distinguish the regime where coherent and incoherent phonons dominate. The MFPs and MFP‐dependent accumulative thermal conductivity are plotted in Figure 2b,c (*N* = 1), and Figure 2e,f (*N* = 5). The markers in Figure 2b,e are colored using the same scheme as in Figure 2a,d. The results suggest that phonons in the coherent regime [i.e., MFP ≥ *d_SL_*/2 (dashed orange lines)] formed by wave interferences transfer heat primarily via the particle‐like propagation in the population‐channel. In contrast, phonons in the incoherent regime (i.e., MFP < *d_SL_*/2), which are mostly intrinsic to the individual layers, predominantly rely on the wave‐channel for heat transfer. Although this observation initially appears to contradict the wave‐like behavior of coherent transport as defined previously in the literature [6, 18, 51], this contradiction can be reconciled by the following explanation. When its MFP exceeds the interface spacing, a phonon crossing an interface in a superlattice does not fully decay and retains partial phase information, thereby enabling coherence between the intrinsic phonons of the individual layers. In our calculations, the superlattice unit cells inherently include coherent phonons, allowing them to contribute to the population‐channel thermal transport due to their large MFPs. This assertion is supported by the observation that the phonon modes that contribute mainly through the population channel have MFPs larger than *d_SL_*/2 (green circles in Figure 2b,e). Conversely, a phonon with a MFP shorter than the interface spacing is scattered before reaching an interface. In this case, the intrinsic phonons of the individual layers are mostly relevant. As a result, these short‐MFP phonons predominately facilitate heat transfer via the wave‐channel, as their wave packets are unable to propagate through the interfaces. These phonon modes have MFPs smaller than *d_SL_*/2 (blue circles in Figure 2b,e). It is also noteworthy that the red circles, which indicate modes that have contributions from both channels, mostly fall in between the Ioffe‐Regel spatial limit (purple dashed line, defined as the average interatomic distance [35]) and *d_SL_*/2, indicating that they are near the crossover between coherent and incoherent behavior.

**FIGURE 3:**
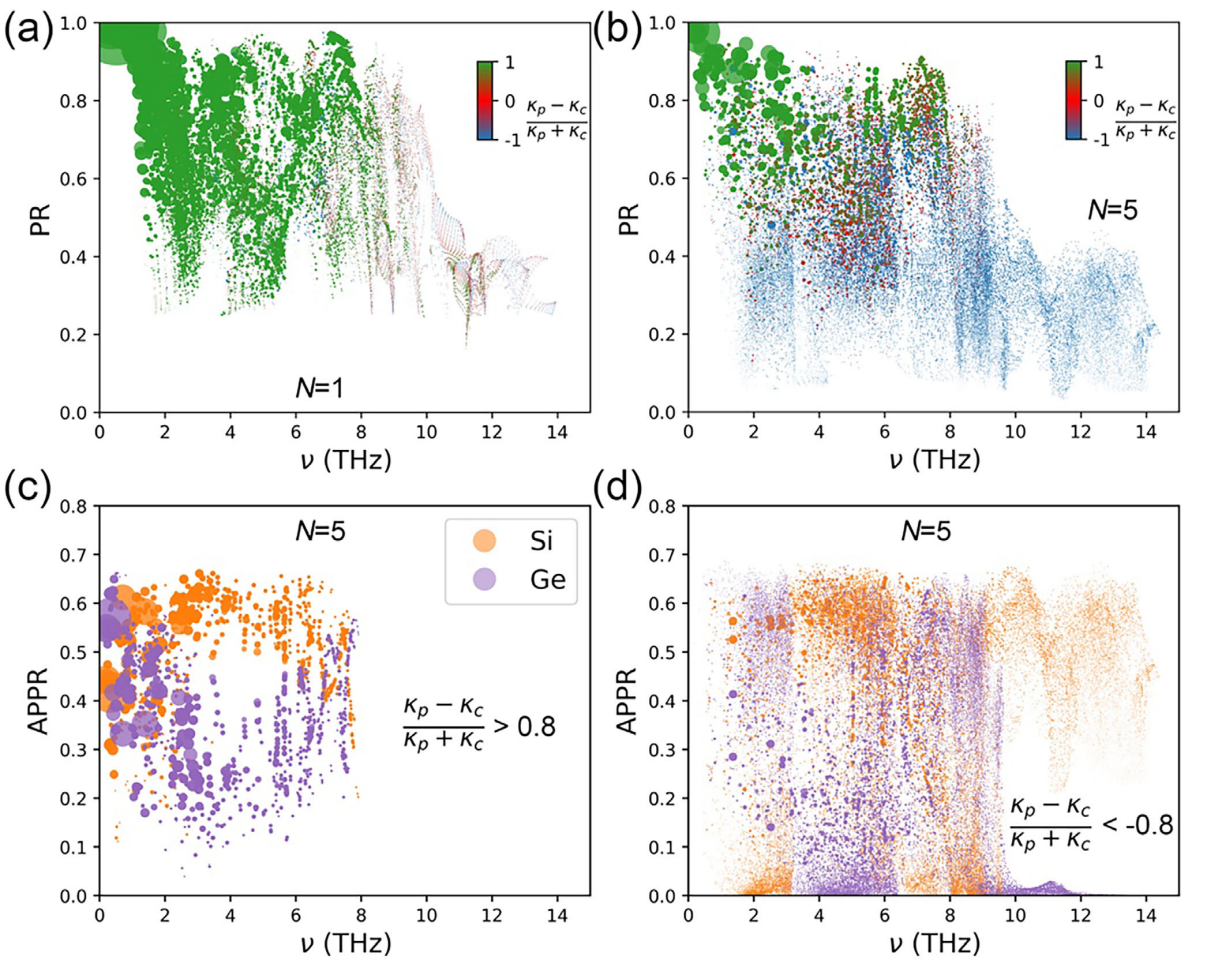
Phonon localization properties of Si[*N*]Ge[*N*] superlattices. Participation ratio (PR) as a function of frequency for the (a) *N*  =  1 and (b) *N*  =  5 superlattices. The color and size of the circles are defined in the Figure 2 caption. (c) and (d) contain the APPR as a function of frequency for the *N*  =  5 superlattice. The orange circles indicate Si atoms and purple circles denote Ge atoms, with the size of the circle representing the thermal conductivity contribution of each phonon mode. (c) APPR for the “green” phonon modes in (b) satisfying the condition (κ_*p*_ − κ_*c*_)/(κ_*p*_ + κ_*c*_) > 0.8; (d) APPR for the “blue” phonon modes in (b) satisfying the condition (κ_*p*_ − κ_*c*_)/(κ_*p*_ + κ_*c*_) < ‐0.8.

To explore the spatial localization of the coherent and incoherent phonons, we computed and plotted the participation ratios (PR) and atomic projected participation ratios (APPR) [52] in Figure 3 (see Methods). This approach has also been applied in studies investigating LJ superlattices [34] and aperiodic Si/Ge superlattices [32]. A comparison of Figure 3a,b reveals that the *N* = 5 superlattice exhibits generally more localized modes with lower PRs than the *N*  =  1 superlattice. Moreover, propagative modes, which are characterized by large PRs [53, 54], transfer heat primarily through the population‐channel (i.e., green circles). Localized modes, on the other hand, are identified by small PRs and contribute dominantly to the wave‐channel thermal conductivity (i.e., blue circles). The APPR distributions of the “green” modes [i.e., (κ_*p*_ − κ_*c*_)/(κ_*p*_ + κ_*c*_) > 0.8] and “blue” modes [i.e., (κ_*p*_ − κ_*c*_)/(κ_*p*_ + κ_*c*_) < ‐0.8] in the 𝑁 = 5 superlattice are shown in Figure 3c,d. The “red” modes are displayed in Figure S3. The “green” modes plotted in Figure 3c involve both Si and Ge atoms, demonstrating the existence of phonon coherence. In contrast, a large portion of “blue” modes plotted in Figure 3d are dominated by vibrations from either Si or Ge atoms, and display a strong spatial confinement effect. These observations reinforce that coherent and incoherent phonons are primarily responsible for population‐ and wave‐channel thermal transport, respectively. We note that some “blue” modes are also formed by vibrations from both Si and Ge atoms, implying that coherent phonons can still appear within the MFP‐determined incoherent regime, and therefore, a strict criterion for distinguishing coherent from incoherent phonons at the mode level may not exist.

### 2.3 Interfacial Vibrational Properties and Their Impact on Phonon Transport

**FIGURE 4:**
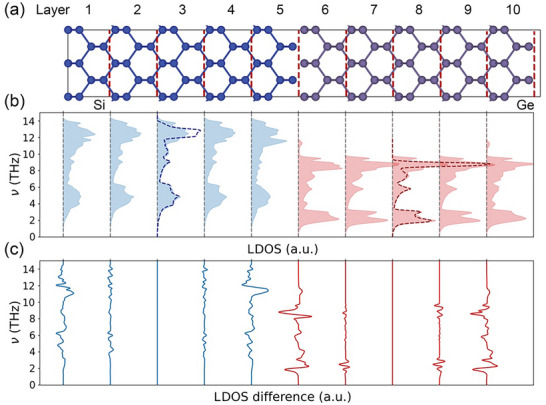
Local vibrational properties of the Si[5]Ge[5] superlattice. (a) Schematic diagram of the superlattice, which is uniformly divided into ten layers along the cross‐plane direction. (b) LDOS of each layer (blue for Si layers, and red for Ge layers); the blue (red) dashed line in the third (eighth) layer represents the DOS of bulk Si (Ge) with a lattice constant at 5.61 Å. (c) Relative differences in the LDOS compared against the reference layers, third layer for Si and eighth layer for Ge.

The Si[5]Ge[5] superlattice is further examined to probe the characteristics of the interfacial vibrational modes, which occur adjacent to the interfaces, and their connection to phonon transport. As shown in Figure 4a, we divided the superlattice into ten layers, each with a thickness of 5.61 Å along the cross‐plane direction. The vibrational properties of atoms in the third and eighth layers are postulated to be similar to those in bulk Si and Ge. Because the second‐order force constants used for the phonon calculations have an interaction radius of 10 Å, Si atoms in the third layer do not interact with any Ge atoms. The same situation applies to the Ge atoms in the eighth layer. This postulation is verified by calculating the local density of states (LDOS) [55, 56, 57] for the third and eighth layers, as shown in Figure 4b. In the 0–8 THz range, the results are in close agreement with the DOS of bulk Si and Ge calculated with the same lattice constant of 5.61 Å. The discrepancies observed at higher frequencies are likely due to slight shifts in the atomic equilibrium positions caused by the lattice mismatch between Si and Ge in the superlattice. Given that the phonon modes in the range of 0–8 THz contribute 89% of the total thermal conductivity of the *N*  =  5 superlattice (Figure S2b), the LDOS of the third and eighth layers are treated as a reference for comparison with the other layers. The LDOS in the layers closest to the interfaces (i.e., first, fifth, sixth, and 10th) deviates noticeably from those of the reference layers (Figure 4c), consistent with previous findings [56, 58]. In contrast, the LDOS for the second, fourth, seventh, and ninth layers shows smaller deviations. Interestingly, the LDOS of the sixth and 10th layers, which contain Ge atoms, display a small peak at approximately 11 THz. This peak cannot originate from the intrinsic Ge vibrations—limited to below 10 THz—and is, instead, attributed to the excitation by Si atoms at the interface. Similar LDOS‐based analysis has also been previously applied to interpret interfacial thermal conductance calculations [59, 60].

**FIGURE 5:**
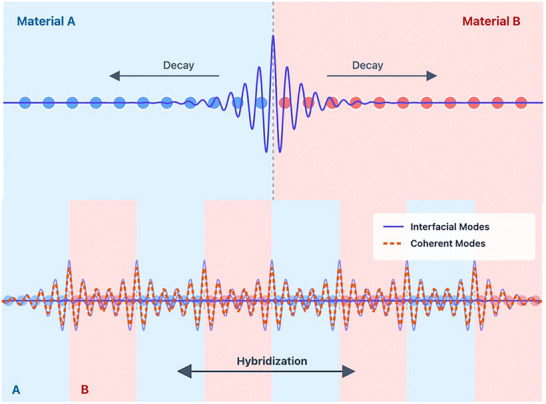
The connection between interfacial modes and coherent modes. The upper panel features an interfacial mode confined to the interface and decaying on both sides, while the lower panel illustrates the emergence of coherent modes arising from the coupling of interfacial modes at neighboring interfaces.

**FIGURE 6:**
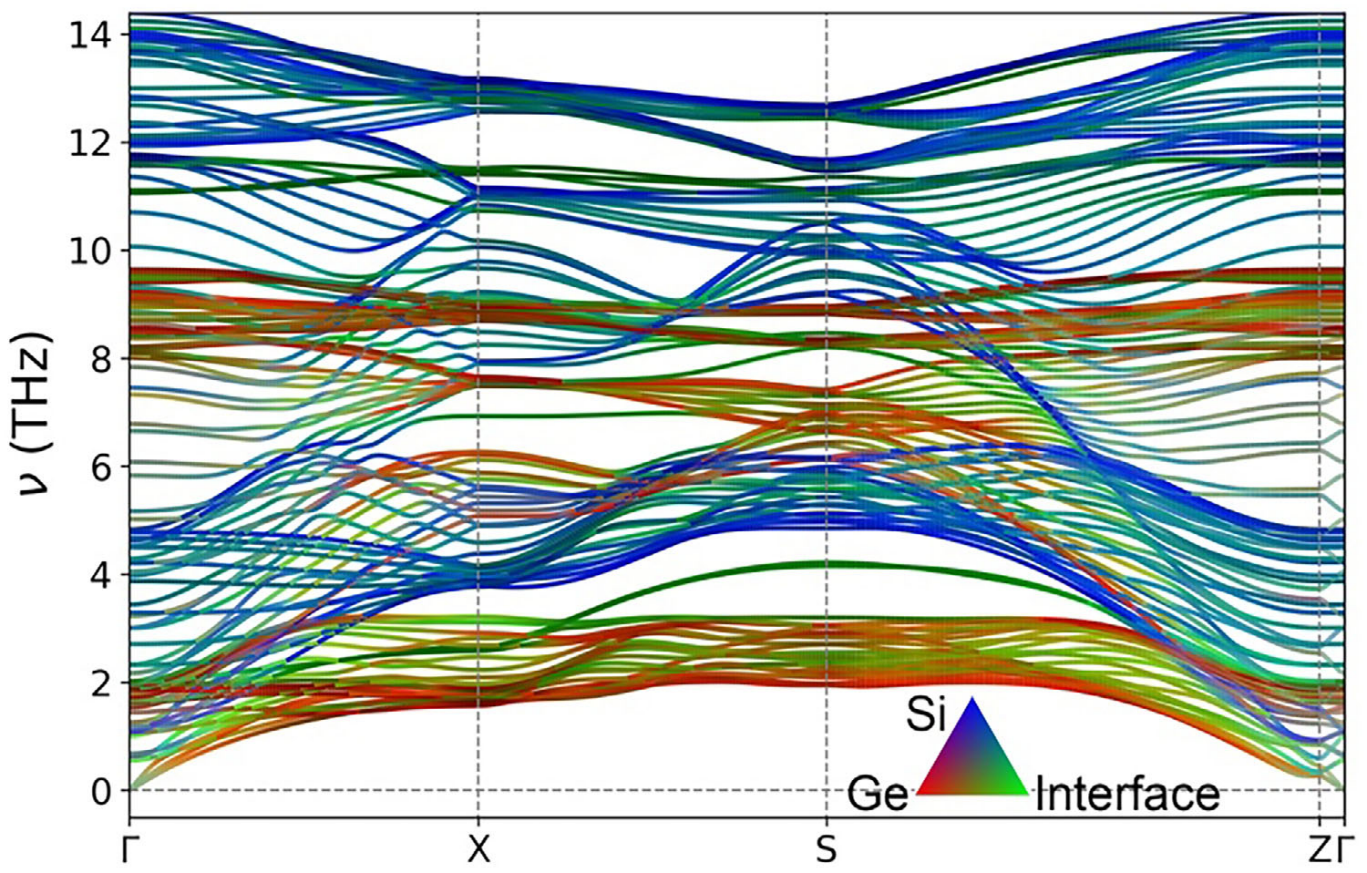
Correlation between phonon dispersion and local vibrational characteristics in the Si[5]Ge[5] superlattice. The color coding represents the proportion of the projected PR in each phonon mode participated by the “intrinsic Ge” layers (layers 7–9, red), the “intrinsic Si” layers (layers 2–4, blue), and the “interface” layers (layers 1, 5, 6, 10, green).

The emergence of coherent phonons in a superlattice arises from the wave interference between the intrinsic phonons of the individual layers at the interface. Specifically, previous studies have shown that variations of the bonding environment near the interface give rise to new interfacial phonon modes that are spatially localized around the interface and decay into both sides [55, 60]. The pronounced LDOS differences in the first, fifth, sixth, and 10th layers relative to the reference layers directly signal the formation of interfacial modes. These localized interfacial modes associated with neighboring interfaces could hybridize across the periodic structure, leading to the formation of Bloch‐like coherent phonons extending throughout the superlattice (see Figure 5). Therefore, the coherent phonon modes are primarily enabled by atomic vibrations near the interface. To investigate these coherent phonon modes in detail, the ten layers of the Si[5]Ge[5] superlattice structure are regrouped into three categories based on the similarities in their LDOS: “interface” layers (first, fifth, sixth, and 10th layers), “intrinsic Si” layers (second, third, and fourth layers), and “intrinsic Ge” layers (seventh, eighth, and nineth layers). Their projected PR mapped onto the phonon dispersion is plotted in Figure 6 (See Methods). The results depict that coherent phonons enabled by atomic vibrations in the “interface” layer (green) are distributed throughout the frequency domain. Notably, these modes are largely confined within the envelopes formed by “intrinsic Si” (blue) phonons and “intrinsic Ge” (red) phonons. A significant increase in the number of coherent phonon modes is observed where the envelope exhibits strong dispersion, particularly along the Γ–X, S–Z, and Z–Γ paths, implying that coherent phonons typically possess higher group velocities than incoherent phonons. This behavior enhances the ability of coherent phonons to transfer heat through the population‐channel, consistent with the analysis from Figure 2.

## 3 Conclusions

We employed the WTE to explore the phonon transport mechanism in two superlattices with smooth interfaces, Ar[*N*]hAr[*N*] and Si[*N*]Ge[*N*]. By treating one period of the superlattice as a unit cell, both systems exhibit a minimum thermal conductivity as a function of period thickness. This result resolves the long‐standing challenge of applying the phonon BTE to model superlattices, which required the addition of a phonon‐boundary scattering to capture the minimum. Moreover, we elucidated the relationship between two wave‐related phonon phenomena in superlattices (i.e., coherence between intrinsic phonons of the individual layers and phonon wave‐channel transport). Our findings reveal that coherent phonons predominantly contribute to the population‐channel thermal conductivity, whereas incoherent phonons primarily contribute to wave‐channel thermal conductivity. As a case study, the Si[5]Ge[5] superlattice reveals that coherent phonons enabled by interface‐localized atomic vibrations have high group velocities, and therefore predominantly participate in population‐channel thermal transport. This study successfully extends the WTE framework to smooth‐interface superlattice systems, offering theoretical guidance for thermal management in relevant materials and devices, and providing new insights into interfacial thermal transport.

## 4 Methods

### 4.1 Construction of Superlattice Models

The crystalline Ar structure was initially optimized using the conjugate gradient algorithm implemented in LAMMPS [61], with interatomic interactions modeled by the Lennard–Jones (LJ) empirical potential and a cutoff distance of 8.5 Å [62]. Due to the identical lattice constants of crystalline Ar and hAr, there is no lattice mismatch in the Ar[*N*]hAr[*N*] superlattices, allowing models with various period thicknesses to be constructed by directly combining the optimized crystalline Ar and hAr structures.

For the Si[*N*]Ge[*N*] superlattices, structural optimizations were performed using the Quantum Espresso package based on density functional theory [63, 64, 65]. The superlattice with a single unit cell, composed of one Si atomic layer and one Ge atomic layer with a smooth interface, was initially employed for the variable‐cell optimization. The exchange‐correlation interaction is described by the Perdew–Burke–Ernzerhof parametrization of the generalized gradient approximation with the ultrasoft pseudopotentials from PSlibrary [66]. The kinetic energy cutoff was set to 50 Ry for the wave function and 400 Ry for the charge density. In terms of the Brillouin zone integration, a 8 × 8 × 8 Monkhorst–Pack grid with a (1, 1, 1) shift was used for the ***k***‐point sampling, where ***k*** is the electron wave vector. During the optimization process, the energy convergence threshold was set to 10^−6^ Ry, the force convergence standard was set to 10^−5^ Ry/Bohr, and the pressure of the unit cell was less than 0.1 kbar to ensure that the system reached a fully relaxed state. Based on the optimized short‐period superlattice, larger‐period superlattices were constructed, ensuring that the atomic arrangement and interfaces remained unchanged.

### 4.2 Compressive Sensing Lattice Dynamics and Thermal Conductivity Calculations

Due to the substantially larger size of superlattice unit cells compared to those of conventional crystals, calculating their interatomic force constants is computationally demanding, limiting the feasibility of traditional methods such as density functional perturbation theory or the finite difference approach. We employed the compressive sensing lattice dynamics (CSLD) method [67] to extract force constants. Compared to the traditional Taylor expansion method with least‐squares fitting, CSLD significantly reduces the number of finite‐displacement supercells needed for force calculations. For each force calculation, ten supercells with dimensions exceeding 20 Å in all directions are generated. Atomic displacements of 0.01 and 0.03 Å are applied for the second‐ and third‐order force constant calculations. For the Ar[*N*]hAr[*N*] superlattices, the effective radius threshold of the second‐order force constant was set to 8.5 Å to match the LJ potential function cutoff, and that for the third‐order force constant was set to 5.5 Å. In the case of the Si[*N*]Ge[*N*] superlattices, the effective radius thresholds of the second‐ and third‐order force constants were set to 10.0 and 6.0 Å.

Once the interatomic force constants were obtained, the phonon population‐channel thermal conductivity was computed using the ShengBTE package [68], while the phonon wave‐channel contribution was evaluated with an in‐house code. A fine ***q***‐grid, where ***q*** is the phonon wave vector, with a spacing of approximately 0.05 Å^−1^ is employed to ensure the convergence of the thermal conductivity. The ***q***‐grid size convergence test for the thermal conductivity of Si[1]Ge[1] superlattice with a period thickness at 11.22 Å is presented in Figure S4. Previous calculations indicate that phonon renormalization and four‐phonon scattering have a negligible impact on the thermal conductivity of bulk Si and bulk Ge at 300 K, therefore, these effects are neglected to reduce computational cost [69, 70].

### 4.3 Calculations of Participation Ratio and Projected Participation Ratio

The PR is defined as [52]:

(2) $${\mathrm{PR}}_{qs}={\left(\sum_{b}^{N_{b}}\frac{{\left|e_{b,qs}\right|}^{2}}{m_{b}}\right)}^{2}\;/N_{b}\sum_{b}^{N_{b}}\frac{{\left|e_{b,qs}\right|}^{4}}{m_{b}^{2}}$$

where *s* denotes the phonon branch, *b* indexes the atoms, ***e*** is the phonon eigenvector, *m* is the atomic mass, and *N_b_* is the number of atoms in the unit cell. The PR can be projected to the contribution from a structural unit (e.g., only one atom type) by [52]

(3) $${\mathrm{PR}}_{qs}=\sum_{b}^{N_{u}}\frac{{\left|e_{b,qs}\right|}^{2}}{m_{b}}\;/{\left(N_{b}\sum_{b}^{N_{u}}\frac{{\left|e_{b,qs}\right|}^{4}}{m_{b}^{2}}\right)}^{\frac{1}{2}}$$

where *N_u_* refers to the number of atoms in the corresponding structural unit. In computing the APPR for Si and Ge atoms (Figure 3c,d), *b* runs over Si and Ge atoms in the unit cell, respectively. For the projected PR associated with the “interface,” “intrinsic Si,” and “intrinsic Ge” layers (Figure 6), *b* is summed over the atoms contained within each layer. In Figure 6, the color‐coded phonon dispersion is constructed using the RGB color code, where each component corresponds to the proportion of the three projected PRs in each phonon mode.

## Author Contributions

J.Y.: methodology, software, formal analysis, investigation, data curation, writing – original draft, and visualization. J.Y.Z.: software, data curation. A.J.H.M.: resources and writing – review and editing. W.‐L.O.: conceptualization, methodology, resources, writing – review and editing, supervision, project administration, and funding acquisition.

## Conflicts of Interest

The authors declare no conflicts of interest.

## Supporting information




**Supporting File**: advs74039‐sup‐0001‐SuppMat.docx

## Data Availability

The data that support the findings of this study are available in the supplementary material of this article.
