# Supplementary material for: Two Distinct Phonon Wave Effects Control Thermal Transport across the Coherent–Incoherent Regime in Superlattices
Source: Adv Sci (Weinh). 2026 Feb 4;13(19):e17251. doi: 10.1002/advs.202517251 (PMC13045394; doi:10.1002/advs.202517251)
Supplement: Supplementary file 1 — Supporting File: advs74039‐sup‐0001‐SuppMat.docx [file ADVS-13-e17251-s001.docx]

**Two Distinct Phonon Wave Effects Control Thermal Transport Across the Coherent–Incoherent Regime in Superlattices**

Jin Yang^1^, Jingyi Zhu^1^, Alan J. H. McGaughey^2^, Wee-Liat Ong^1, 3, *^

^1^ ZJU-UIUC Institute, College of Energy Engineering, Zhejiang University, Jiaxing, Haining, Zhejiang 314400, China

^2^ Department of Mechanical Engineering, Carnegie Mellon University, Pittsburgh, Pennsylvania 15213, USA

^3^ State Key Laboratory of Clean Energy Utilization, Zhejiang University, Hangzhou, Zhejiang 310027, China

* Corresponding author

Tel: 0571-87572500. Email: [weeong@intl.zju.edu.cn](mailto:weeong@intl.zju.edu.cn)

## Supplemental Note A: Details of Molecular Dynamics Simulations

The molecular dynamics simulations of the Ar[$N$]hAr[$N$] superlattices were performed using LAMMPS^1^, with interatomic interactions modeled by the Lennard-Jones (LJ) empirical potential and a cutoff distance of 8.5 Å^2^. A time step of 1 fs was employed and periodic boundary conditions were applied in all three spatial dimensions. To prevent interactions between periodic images, fixed regions longer than 25 Å were placed at both ends of the system. The system was first relaxed for 2 ns in the *NVT* ensemble to stabilize the temperature at 20 K, followed by a 2 ns equilibration in the *NVE* ensemble, resulting in a final system pressure of 0.06 GPa. Non-equilibrium molecular dynamics simulations (NEMD) were then conducted for 30 ns to ensure that the system reached a steady state. A Langevin heat bath with the same length as the fixed region was used to maintain the hot and cold reservoirs at temperatures of 22 K and 18 K. The heat flow and temperature gradient data from a further 10 ns were taken to calculate the thermal conductivity from the Fourier Law. The system cross section size was 21 Å×21 Å. The superlattice models with different period thicknesses have different total lengths to ensure convergence of the thermal conductivity. The period thicknesses-dependent thermal conductivity and the length-dependent convergence of the thermal conductivity for the Ar[0.5]hAr[0.5] superlattice are plotted in Figure S1. The statistical uncertainty of each MD data point in Figure S1a comes from the standard deviation of three independent simulations. The converged values in Figure S1a correspond to the second largest system, with a total length exceeding 1100 Å.


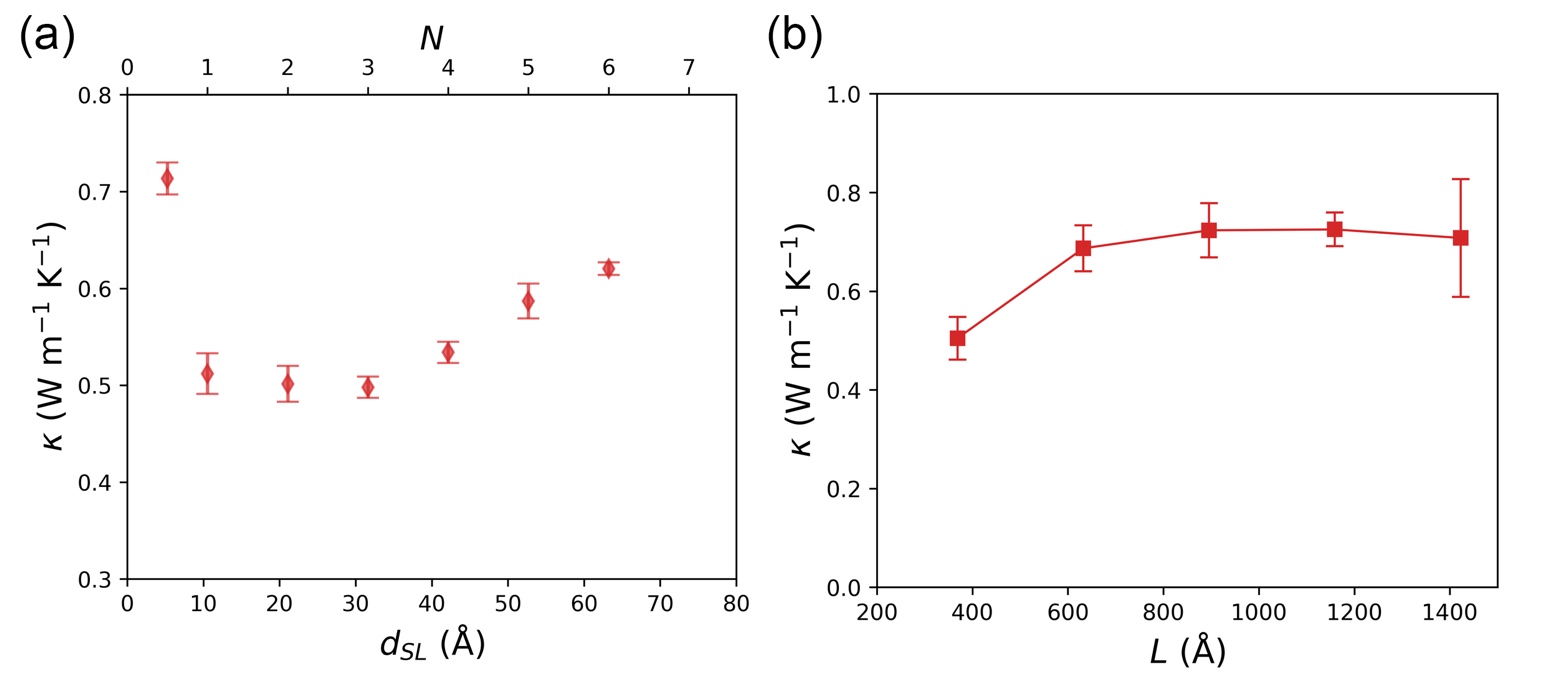


Figure S1. (a) Cross-plane (i.e., along the Cartesian *z*-direction) NEMD thermal conductivity of Ar[*N*]hAr[*N*] superlattices with different period thickness. (b) Length convergence of the NEMD thermal conductivity along the cross-plane direction for the Ar[0.5]hAr[0.5] superlattice, which has a period thickness at 5.27 Å.


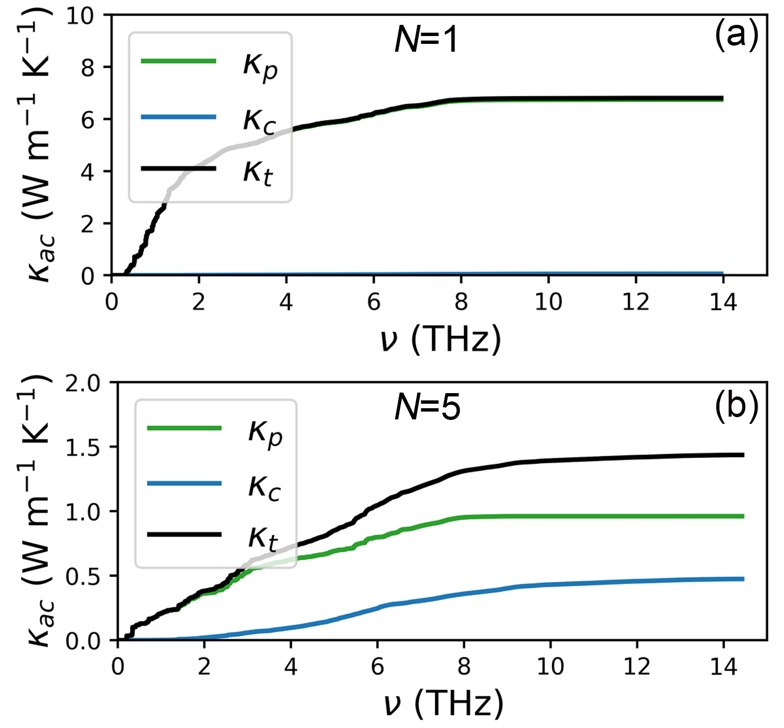


Figure S2. Frequency-dependent accumulative thermal conductivity for the Si[*N*]Ge[*N*] superlattices with (a) $N$ = 1 and (b) $N$ = 5.


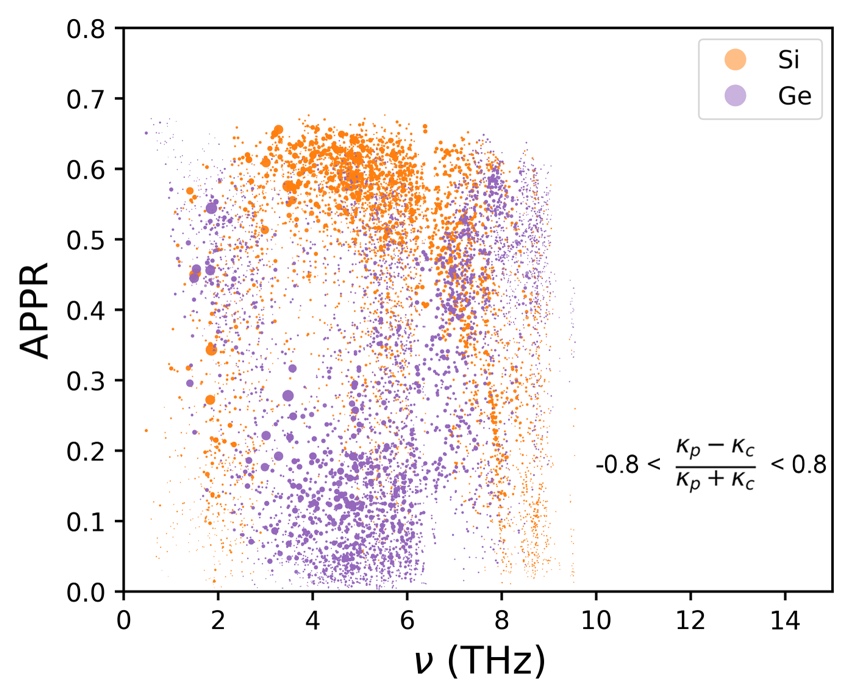


Figure S3. Atomic projected participation ration for the “red” phonon modes satisfying the condition -0.8 < ($\kappa_{p}-\kappa_{c})/(\kappa_{p}+\kappa_{c})$ < 0.8 of the Si[5]Ge[5] superlattice.


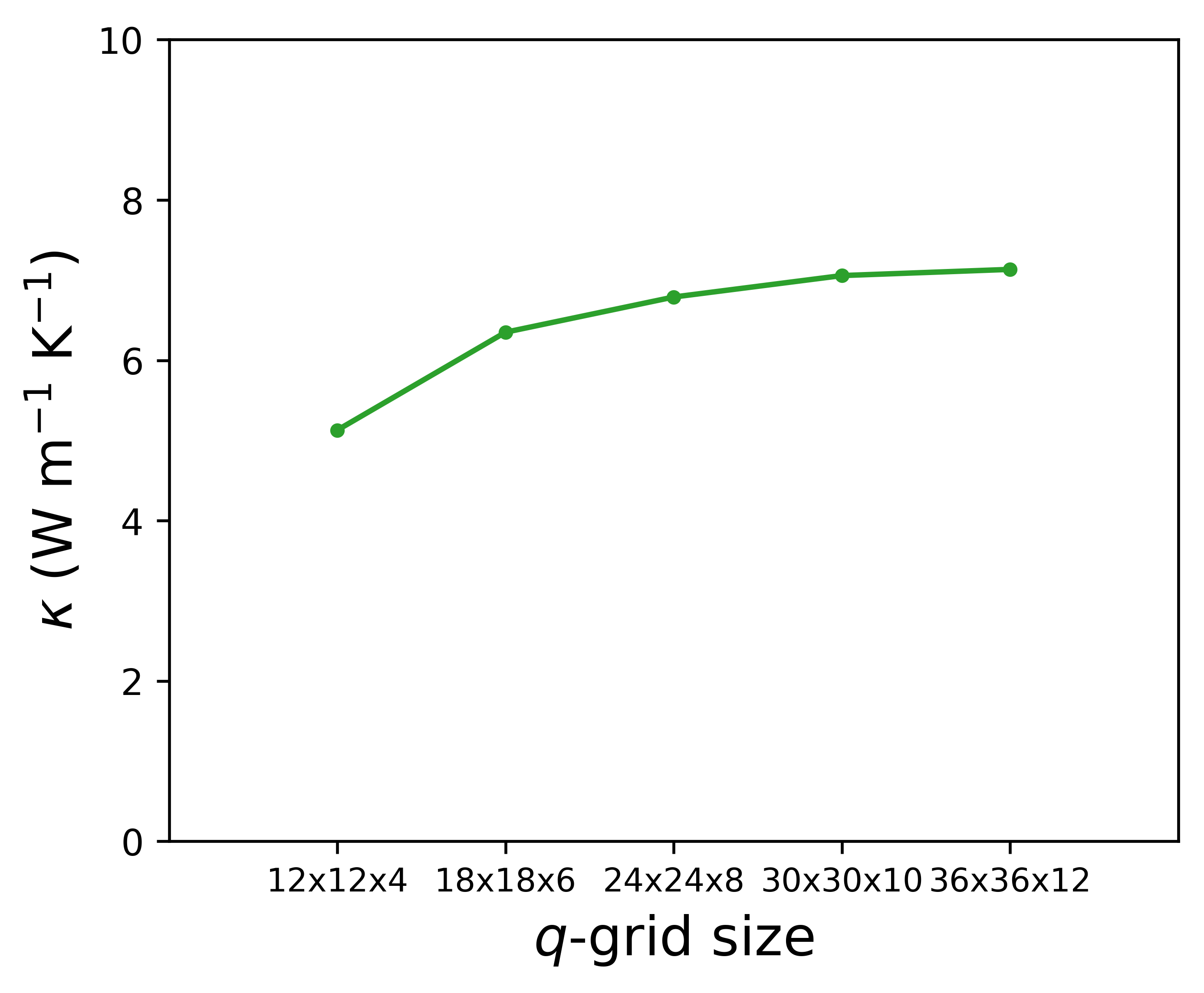


Figure S4. $\boldsymbol{q}$-grid convergence test of the population-channel thermal conductivity along the cross-plane direction for the Si[1]Ge[1] superlattice, which has a period thickness at 11.22 Å. As the coherence-channel contributions are minimal, they are not displayed. The $\boldsymbol{q}$-grid of 30×30×10 corresponds to a spacing at 0.05 Å^-1^.

**References**

1 A. P. Thompson, H. M. Aktulga, R. Berger, D. S. Bolintineanu, W. M. Brown, P. S. Crozier, P. J. In ’T Veld, A. Kohlmeyer, S. G. Moore, T. D. Nguyen, R. Shan, M. J. Stevens, J. Tranchida, C. Trott and S. J. Plimpton, *Comput. Phys. Commun.*, 2022, **271**, 108171.

2 T. Ikeshoji and B. Hafskjold, *Mol. Phys.*, 1994, **81**, 251–261.
